# Revocable identity-based proxy re-signature against signing key exposure

**DOI:** 10.1371/journal.pone.0194783

**Published:** 2018-03-26

**Authors:** Xiaodong Yang, Chunlin Chen, Tingchun Ma, Jinli Wang, Caifen Wang

**Affiliations:** 1 College of Computer Science and Engineering, Northwest Normal University, Lanzhou, Gansu, China; 2 State Key Laboratory of Cryptology, Beijing, China; King Saud University, SAUDI ARABIA

## Abstract

Identity-based proxy re-signature (IDPRS) is a novel cryptographic primitive that allows a semi-trusted proxy to convert a signature under one identity into another signature under another identity on the same message by using a re-signature key. Due to this transformation function, IDPRS is very useful in constructing privacy-preserving schemes for various information systems. Key revocation functionality is important in practical IDPRS for managing users dynamically; however, the existing IDPRS schemes do not provide revocation mechanisms that allow the removal of misbehaving or compromised users from the system. In this paper, we first introduce a notion called revocable identity-based proxy re-signature (RIDPRS) to achieve the revocation functionality. We provide a formal definition of RIDPRS as well as its security model. Then, we present a concrete RIDPRS scheme that can resist signing key exposure and prove that the proposed scheme is existentially unforgeable against adaptive chosen identity and message attacks in the standard model. To further improve the performance of signature verification in RIDPRS, we introduce a notion called server-aided revocable identity-based proxy re-signature (SA-RIDPRS). Moreover, we extend the proposed RIDPRS scheme to the SA-RIDPRS scheme and prove that this extended scheme is secure against adaptive chosen message and collusion attacks. The analysis results show that our two schemes remain efficient in terms of computational complexity when implementing user revocation procedures. In particular, in the SA-RIDPRS scheme, the verifier needs to perform only a bilinear pairing and four exponentiation operations to verify the validity of the signature. Compared with other IDPRS schemes in the standard model, our SA-RIDPRS scheme greatly reduces the computation overhead of verification.

## Introduction

A digital signature provides security services such as data integrity, authentication and non-repudiation; therefore, it is one a key technology to ensure information security. In particular, digital signatures can be combined with passwords [1, 2], smart cards [3], biometrics [4], chaotic parallel keyed hash functions [5] and other technologies to achieve identity authentication of parties in communication. Consequently, they have practical applications in systems such as smart cities [6], body area networks [7], the Internet of Things [8] and ubiquitous networks [9]. To meet the security needs of different practical scenarios, researchers have proposed a series of digital signature variants. For instance, a blind signature can effectively protect the content of signed messages; therefore, it is applied to e-commerce, e-voting, e-currency and other systems to protect participants’ interests [10, 11]. A group signature allows any member of a group to sign messages on behalf of the entire group anonymously; therefore, group signatures have been used to protect the privacy and authenticate the identity of vehicles in vehicular ad-hoc networks [12]. A ring signature is a type of group signature with no administrator that can be used in cloud computing and block-chains to achieve unconditional anonymity to protect a user’s privacy [13, 14]. In many scenarios, we need, for example, to verify Alice’s signature, but we do not know her public key or her public key has expired. We want to transform Alice’s signature into Bob’s signature, because Bob’s public key is available. To achieve this transformation, Blaze et al. [15] introduced the concept of a proxy re-signature (PRS). In a PRS scheme, a semi-trusted proxy is allowed to transform Alice’s signature on a message into Bob’s signature on that same message. However, the proxy cannot independently generate a valid signature on any message on behalf of Alice or Bob. Instead, the proxy re-signature functions as a signature conversion method; consequently, it has been widely used in areas such as cross-domain authentication, digital rights management, privacy preservation and auditing in cloud computing environments.

Ateniese and Hohenberger [16] provided a security model for PRS and presented two concrete schemes in 2005: one is multi-use and the other is single-use. Generally speaking, in a multi-use PRS scheme, a proxy can transform a signature from Alice to Bob into a signature from Bob to Carol, but a proxy cannot further convert a transformed signature in a single-use PRS scheme. In the proxy re-signature field, a multi-use PRS is more useful than is a single-use PRS. After Ateniese and Hohenberger’s seminal work [16], other PRS schemes with special properties were proposed, including the universally composable secure PRS [17], the certificateless PRS [18] and the threshold PRS [19]. In particular, Shao et al. [20] proposed an identity-based proxy re-signature (IDPRS) scheme to address the problem of certificate management in traditional PRS schemes. In IDPRS, the user’s email address or other unique identifying information is used as the public key, and the user’s private key is generated by a trusted private key generator (PKG). IDPRS allows a semi-trusted proxy to convert a signature under one identity to another signature under another identity on the same message, but the proxy is unable to produce any signature on behalf of any of these two identities. As a result, IDPRS eliminates the requirement for certificates and simplifies key management, but challenges still remain to be addressed in practical applications.

Shao et al. [20] proposed the first IDPRS scheme in the standard model. Later, Feng et al. [21] proposed a secure IDPRS scheme, but it was not multi-use. Hu et al. also [22] presented an IDPRS scheme without random oracles, but its security relies on a strong difficult problem assumption. Tian [23] proposed an IDPRS scheme from lattices in the random oracle model, but the size of the signature was relatively large and its practicality was poor. In addition, Wang et al. [24] constructed two server-aided proxy re-signature schemes that were provably secure in the random oracle model, but the second scheme cannot resist a collusion attack from the server and a malicious proxy. Moreover, Canetti et al. [25] found that the provably secure scheme in the random oracle model might be insecure in reality when the random oracle is instantiated by a specific hash function. Therefore, it is of practical significance to construct secure RIDPRS schemes in the standard model.

The existing IDPRS schemes do not consider the problem of user revocation [20–23]. A revocation mechanism is essential for practical identity-based cryptosystem [26]. If a user’s key is compromised or a user’s authorization expires, that user needs to be revoked from the system. When users have been revoked, they should no longer be able to use their previous private keys to gain access to sensitive data or to generate valid digital signatures. Researchers have designed a series of revocable cryptographic schemes to achieve user revocation in identity-based settings [27–29]. The main idea behind these schemes is that the PKG periodically updates the private keys of non-revoked users. Tsai et al. [30] proposed a revocable identity-based signature scheme in the standard model, in which a user’s signing key consists of a long-term secret key and a periodically changed update key. However, Tsai et al.’s scheme [30] is insecure against a signing key exposure attack: an adversary can obtain a fixed secret key from a compromised signature key and then combine it with subsequent update keys to forge the signature of any message. Lian et al. [31] proposed a revocable attribute-based signature scheme, but Wei et al. [32] revealed that Lian et al.’s scheme is vulnerable to signing key exposure. If a signature scheme can resist the signing key exposure attack, then the users store their secret keys on physical devices with relatively high security levels, while the update keys can be stored on devices with relatively low security levels (such as mobile phones, smart cards, etc.). However, to the best of our knowledge, no identity-based proxy re-signature scheme with a user revocation mechanism is available. Therefore, how to design a revocable identity-based proxy re-signature (RIDPRS) scheme is an open and interesting question.

In this paper, we formally define the syntax of RIDPRS and present a security model for RIDPRS against signing key exposure. Based on Shao et al.’s IDPRS scheme [20], we design a bidirectional and multi-use RIDPRS scheme. In the proposed scheme, the PKG’s master secret key is divided into two parts: one part is used to construct the user’s fixed secret key, and the other part is used to generate periodically changed update keys for the user. Only a non-revoked user can re-randomize their secret key and update key to generate a corresponding signing key. Consequently, our scheme can not only effectively revoke a user from the system, but also resist signing key exposure attacks. Furthermore, we introduce a new cryptographic primitive called server-aided revocable identity-based proxy re-signature (SA-RIDPRS), which is particularly suitable for verifiers with limited computing power. SA-RIDPRS allows the verifier to delegate most of the computational work involved in signature verification to a server with powerful computing capabilities; the verifier needs to perform only a small number of computational operations to verify the legitimacy of the signature. In addition, we formalize the security model for SA-RIDPRS. Based on our RIDPRS scheme, we also construct an SA-RIDPRS scheme and prove that the proposed scheme is secure against adaptive chosen message and collusion attacks. The results of our analysis show that our two schemes are bidirectional and multi-use, and they achieve user revocation functionality while maintaining efficiency in terms of computational complexity and storage overhead. In our SA-RIDPRS scheme, the verifier verifies the validity of the signature with minimal computational cost by executing the server-aided verification algorithm in conjunction with a server, which efficiently reduces the computational cost of the verifier.

The rest of this paper is organized as follows. Section 2 reviews some preliminaries used in our schemes. Section 3 presents security notions for RIDPRS and SA-RIDPRS. Section 4 constructs an IDPRS scheme and an SA-RIDPRS scheme and gives their security proof and performance analysis. Finally, Section 5 concludes the paper.

## Preliminaries

In this section, we briefly review bilinear parings and the computational Diffie-Hellman (CDH) assumption.

### Bilinear parings

Let *p* be a prime, *G*_1_ and *G*_2_ be two multiplicative cyclic groups of order *p*, and *g* be a generator of *G*_1_. An efficiently computable bilinear paring *e*: *G*_1_ × *G*_1_ → *G*_2_ is a map with the following properties [16]:

- *Bilinear:* *e*(*g*^*a*^, *g*^*b*^) = *e*(*g*, *g*)^*ab*^ = *e*(*g*^*b*^, *g*^*a*^) for any *a*, *b* ∈ *Z*_*p*_.
- *Non-degenerate:* *e*(*g*, *g*) ≠ 1.

### Complexity assumption

The security of our scheme depends on the hardness of the CDH problem. Let *G*_1_ be a cyclic group of prime order *p*, and *g* be a generator of *G*_1_. Given $\left(g,g^{a},g^{b}\right)\in G_{1}^{3}$, where *a*, *b* ∈ *Z*_*p*_, the CDH problem is to compute *g*^*ab*^ ∈ *G*_1_.

**Definition 1.** We say that the CDH assumption holds if no probabilistic polynomial-time (PPT) algorithm can solve the CDH problem in *G*_1_ with a non-negligible probability [20].

## Formal definition and security model

### Security notions of RIDPRS

An RIDPRS scheme consists of the following eight algorithms (**Setup**, **Extract**, **KeyUp**, **SKGen**, **ReKey**, **Sign**, **ReSign**, **Verify**):

- **Setup**(*λ*) → (*pp*, *msk*): Given a security parameter *λ*, this algorithm outputs public parameters *pp* and a master secret key *msk* of the PKG.
- **Extract**(*pp*, *msk*, *ID*) → *sk*_*ID*_: Given *pp*, *msk* and a user’s identity *ID*, this algorithm outputs a secret key *sk*_*ID*_ of the identity *ID*.
- **KeyUp**(*pp*, *msk*, *ID*, *t*) → *vk*_*ID*,*t*_: Given *pp*, *msk*, an identity *ID* and a time period *t*, this algorithm outputs an update key *vk*_*ID*,*t*_ with respect to identity *ID* and time period *t*.
- **SKGen**(*pp*, *sk*_*ID*_, *vk*_*ID*,*t*_) → *dk*_*ID*,*t*_: Given *pp*, a secret key *sk*_*ID*_ and an update key *vk*_*ID*,*t*_, this algorithm outputs an error symbol ⊥ if the identity *ID* has been revoked during the time period *t*; otherwise, it outputs a signing key *dk*_*ID*,*t*_ on (*ID*, *t*).
- **ReKey**(*pp*, *dk*_*A*,*t*_, *dk*_*B*,*t*_) → *rk*_*A*→*B*,*t*_: Given *pp* and two signing keys (*dk*_*A*,*t*_, *dk*_*B*,*t*_) corresponding to identities (*ID*_*A*_, *ID*_*B*_) at time period *t*, this algorithm outputs a re-signing key *rk*_*A*→*B*,*t*_ of the proxy.
- **Sign**(*pp*, *dk*_*ID*,*t*_, *M*) → *σ*: Given *pp*, a signing key *dk*_*ID*,*t*_ and a message *M*, this algorithm generates a signature *σ* on *M*.
- **ReSign**(*pp*,*rk*_*A*→*B*_, *ID*_*A*_, *t*, *M*, *σ*_*A*_) → *σ*_*B*_: Given *pp*, a re-signing key *rk*_*A*→*B*_ and a signature *σ*_*A*_ on a message *M* with respect to identity *ID*_*A*_ and time period *t*, this algorithm outputs ⊥ if **Verify**(*pp*, *ID*_*A*_, *t*, *M*, *σ*_*A*_) = 0; otherwise, it outputs a signature *σ*_*B*_ on *M* with respect to identity *ID*_*B*_ and time period *t*.
- **Verify**(*pp*, *ID*, *t*, *M*, *σ*) → {0, 1}: Given *pp*, an identity *ID*, a time period *t*, a message *M* and a signature *σ*, this algorithm outputs 1 if *σ* is a valid signature of *M* on (*ID*, *t*); otherwise, it outputs 0.

**Correctness.** Let (*pp*, *msk*) be the output of the algorithm **Setup**(*λ*). For any message *M* and any identities (*ID*_*A*_, *ID*_*B*_), if *σ*_*A*_ = **Sign**(*pp*, *t*, *dk*_*A*,*t*_, *M*) and *σ*_*B*_ = **ReSign**(*pp*,*rk*_*A*→*B*_, *ID*_*A*_, *t*, *M*, *σ*_*A*_), then the conditions **Verify**(*pp*, *ID*_*A*_, *t*, *M*, *σ*_*A*_) = 1 and **Verify**(*pp*, *ID*_*B*_, *t*, *M*, *σ*_*B*_) = 1 must both hold.

The security of an RIDPRS scheme should be existentially unforgeable under adaptive chosen identity and message attacks. Based on the security model of IDPRS in [20] and the security definition of revocable identity-based signature schemes in [30–32], the existential unforgeability of a bidirectional RIDPRS scheme that captures signing key exposure is formally defined by using the following security game between a challenger B and an adversary A:

**Setup:** B executes the algorithm **Setup**(*λ*) to generate public parameters *pp* and the PKG’s master secret key *msk*. Then, B sends *pp* to A.

**Queries:** The adversary A adaptively makes the following queries:

- *Extract-query:* When A asks for a secret key of an identity *ID*, B executes the algorithm **Extract**(*pp*, *msk*, *ID*) and returns the corresponding output *sk*_*ID*_ to A.
- *KeyUp-query:* When A inquires about an update key with respect to an identity *ID* and a time period *t*, B executes the algorithm **KeyUp**(*pp*, *msk*, *ID*, *t*) and returns an update key *vk*_*ID*,*t*_ to A.
- *SKGen-query:* When A asks for a signing key with respect to an identity *ID* and a time period *t*, B first makes an *Extract-query* for *ID* and a *KeyUp-query* for the tuple (*ID*, *t*) to obtain a secret key *sk*_*ID*_ and an update key *vk*_*ID*,*t*_, respectively. Then, B runs the algorithm **SKGen**(*pp*, *sk*_*ID*_, *vk*_*ID*,*t*_) to generate a signing key *dk*_*ID*,*t*_, and sends it to A.
- *ReKey-query:* When A requests a re-signing key of two identities (*ID*_*A*_, *ID*_*B*_) at time period *t*, B first makes the *SKGen-query* on tuples (*ID*_*A*_, *t*) and (*ID*_*B*_, *t*) to obtain the corresponding signing keys *dk*_*A*,*t*_ and *dk*_*B*,*t*_, respectively. Afterwards, B runs the algorithm **ReKey**(*pp*, *dk*_*A*,*t*_, *dk*_*B*,*t*_) to output a re-signing key *rk*_*A*→*B*,*t*_, and then sends it to A.
- *Sign-query:* When A requests a signature on a message *M* with respect to an identity *ID* and a time period *t*, B first makes a *SKGen-query* on tuple (*ID*, *t*) to obtain a signing key *dk*_*ID*,*t*_. Then, B runs the algorithm **Sign**(*pp*, *dk*_*ID*,*t*_, *M*) and returns a signature *σ* on *M* to A.

**Forgery:** The adversary A finally outputs a forged signature σ* on a message M* with respect to an identity ID* and a time period t*. We say that A wins in the above game if the following conditions hold.

1. **Verify**(*pp*, ID*, t*, M*, σ*) = 1.
2. (ID*, t*) has never been queried of the *SKGen-query*.
3. ID* has never been submitted to the *Extract-query*, and (ID*, t*) has never been submitted to the *KeyUp-query*.
4. ID* has never been submitted to the *ReKey-query*.
5. (ID*, t*, M*) has never been queried of the *Sign-query*.

**Definition 2.** A bidirectional RIDPRS scheme is said to be existentially unforgeable against adaptive chosen identity and message attacks if for any polynomial-time adversary A the probability of winning in the above game is negligible.

### Security notions of SA-RIDPRS

An SA-RIDPRS scheme consists of an RIDPRS scheme and a server-aided verification protocol. Due to the weaker computing power, the verifier is unable to perform complicated cryptographic operations. Therefore, the verifier needs to execute an interactive verification protocol to verify the validity of the signature with the help of a server. Specifically, a bidirectional SA-RIDPRS scheme is a tuple of the following ten algorithms (**Setup**, **Extract**, **KeyUp**, **SKGen**, **ReKey**, **Sign**, **ReSign**, **Verify**, **SA-Setup**, **SA-Verify**):

- The **Setup**, **Extract**, **KeyUp**, **SKGen**, **ReKey**, **Sign**, **ReSign** and **Verify** algorithms are the same as those in the RIDPRS scheme described in Section 3.1.
- **SA-Setup**(*pp*) → *V String*: On input public parameters *pp*, this algorithm generates a secret string *V String* that contains the pre-computed information of the verifier.
- **SA-Verify**(*pp*, *V String*, *ID*, *t*, *M*, *σ*) → {0, 1}: On input *pp*, *V String* and a signature *σ* on a message *M* with respect to an identity *ID* and a time period *t*, this algorithm outputs 1 if the server convinces the verifier that *σ* is valid; otherwise, it outputs 0.

The security of an SA-RIDPRS scheme includes both the existential unforgeability of RIDPRS and the soundness of the algorithm **SA-Verify**. Existential unforgeability ensures that an attacker cannot generate a valid signature on a new message, whereas soundness ensures that the server cannot convince a verifier that an invalid signature is valid. The unforgeability of a bidirectional SA-RIDPRS scheme is the same as that of the RIDPRS scheme defined in Section 3.1. Based on the soundness of server-aided verification PRS [24], the soundness of the algorithm **SA-Verify** under adaptive chosen message and collusion attacks is defined by the following security game between a challenger C and an adversary A. In this game, the challenger C acts as a verifier while the adversary A acts as the server. A is allowed to collude with the signer or the proxy; therefore, A can generate or transform the signature of any message. The goal of A is to convince C that an invalid signature is valid. C and A interact as follows:

**Setup:** C executes the **Setup** and **SA-Setup** algorithms to generate the public parameters *pp* and the string *V String*, respectively. Then, C sends *pp* to A.

**Queries:** A can adaptively make a number of server-aided verification queries to C. For each query on (*ID*_*i*_, *t*_*i*_, *M*_*i*_, *σ*_*i*_), C runs the algorithm **SA-Verify** with A and then returns the corresponding output to A as a response.

**Forgery:** The adversary A eventually outputs an identity ID*, a time period t*, a message M* and a string σ*. Let *Ω*_M*_ be the set of all valid signatures on M*, where σ* ∉ *Ω*_M*_. If **Verify**(*pp*, ID*, t*, M*, σ*) = 0 and **SA-Verify**(*pp*, *V String*, ID*, t*, M*, σ*) = 1, which means that A has convinced C that σ* is a valid signature on M* with respect to the tuple (ID*, t*), then the adversary A wins the game.

**Definition 3.** The algorithm **SA-Verify** is soundness if the probability that any polynomial-time adversary A wins in the above game is negligible.

**Definition 4.** If an RIDPRS scheme is existentially unforgeable against adaptive chosen identity and message attacks, and the algorithm **SA-Verify** is soundness, then the resulting SA-RIDPRS scheme is said to be secure against adaptive chosen message and collusion attacks.

## Our constructions

In this section, we first construct a bidirectional RIDPRS scheme based on the IDPRS scheme of Shao et al. [20]. Then, we extend it to the SA-RIDPRS scheme. Moreover, we provide security proofs and performance analyses for the proposed schemes.

### Bidirectional and revocable ID-based proxy re-signature scheme

#### Description

In our RIDPRS scheme, we assume that the length of the identity and the length of the message are *n*_*u*_-bit and *n*_*m*_-bit strings, respectively. We can achieve these by two collision-resistant hash functions $H_{1}:{\left\{0,1\right\}}^{*}\to {\left\{0,1\right\}}^{n_{u}}$ and $H_{2}:{\left\{0,1\right\}}^{*}\to {\left\{0,1\right\}}^{n_{m}}$. The details of our RIDPRS scheme are described as follows:

- **Setup**: Given a security parameter *λ*, the PKG does the following:
  1. Choose two multiplicative cyclic groups *G*_1_ and *G*_2_ of prime order *p*, a generator *g* of *G*_1_ and a bilinear pairing *e*: *G*_1_ × *G*_1_ → *G*_2_.
  2. Select a collision-resistant hash function $H:{\left\{0,1\right\}}^{*}\to {\left\{0,1\right\}}^{n_{v}}$, where *n*_*v*_ is the fixed length of the output of *H*.
  3. Pick four random elements *g*_2_, *u*_0_, *v*_0_, *w*_0_ ∈ *G*_1_ and three random vectors $\vec{u}=\left(u_{i}\right)$, $\vec{v}=\left(v_{j}\right)$ and $\vec{w}=\left(w_{k}\right)$ from *G*_1_, where 1 ≤ *i* ≤ *n*_*u*_, 1 ≤ *j* ≤ *n*_*v*_ and 1 ≤ *k* ≤ *n*_*m*_.
  4. Choose two random integers $\alpha ,\beta \in Z_{p}^{*}$ and compute $g_{1}=g^{\alpha +\beta }$.
  5. Store the master secret key $msk=(g_{2}^{\alpha },g_{2}^{\beta })$ secretly and publish the public parameters $pp=(G_{1},G_{2},e,p,g,g_{1},g_{2},H,u_{0},v_{0},w_{0},\vec{u},\vec{v},\vec{w})$.
  To simplify the expression, for any identity *ID* = ($ID_{1},\ldots ,ID_{n_{u}})\in {\left\{0,1\right\}}^{n_{u}}$, any string *T* = (*T*_1_, …, $T_{n_{v}})\in {\left\{0,1\right\}}^{n_{v}}$ and any message *M* = (*M*_1_, …, *M*_*n*_*m*__) $\in {\left\{0,1\right\}}^{n_{m}}$, we define the following three functions
  $F_{W,1}\left(ID\right)=u_{0}\prod_{i=1}^{n_{u}}{\left(u_{i}\right)}^{ID_{i}}$, $F_{W,2}\left(T\right)=v_{0}\prod_{j=1}^{n_{v}}{\left(v_{j}\right)}^{T_{j}}$ and $F_{W,3}\left(M\right)=w_{0}\prod_{k=1}^{n_{m}}{\left(w_{k}\right)}^{M_{k}}$, respectively.
- **Extract**: Given a user’s identity *ID*, the PKG first chooses a random integer *r*_*ID*_ ∈ *Z*_*p*_ and computes a secret key $sk_{ID}=\left(sk_{ID,1},sk_{ID,2}\right)=(g_{2}^{\alpha }F_{W,1}{\left(ID\right)}^{r_{ID}}$, $g^{r_{ID}})$. Then, the PKG sends *sk*_*ID*_ to the user via a secure channel.
- **KeyUp**: Given an identity *ID* and a time period *t*, the PKG randomly chooses *s*_*ID*_ ∈ *Z*_*p*_, and computes *T*_*ID*_ = *H*(*ID*, *t*) and *vk*_*ID*,*t*_ = (*vk*_*ID*,*t*,1_, *vk*_*ID*,*t*,2_) = $\left(g_{2}^{\beta }F_{W,2}{\left(T_{ID}\right)}^{s_{ID}},g^{s_{ID}}\right)$. Then, the PKG sends an update key *vk*_*ID*,*t*_ to the user via a public channel.
- **SKGen**: On input (*ID*, *t*), if identity *ID* has been revoked within time period *t*, the user is unable to generate a valid signing key because he cannot obtain valid update keys. Otherwise, the user with identity *ID* uses his secret key *sk*_*ID*_ = (*sk*_*ID*,1_, *sk*_*ID*,2_) and update key *vk*_*ID*,*t*_ = (*vk*_*ID*,*t*,1_, *vk*_*ID*,*t*,2_) to generate a signing key using the following steps:
  1. Choose two random integers $r_{ID}^{\prime },s_{ID}^{\prime }\in Z_{p}$.
  2. Compute *T*_*ID*_ = *H*(*ID*, *t*), $dk_{ID,t,2}=sk_{ID,2}\cdot g^{r_{ID}^{\prime }}=g^{r_{ID}+r_{ID}^{\prime }}$, $dk_{ID,t,3}=vk_{ID,t,2}\cdot g^{s_{ID}^{\prime }}=g^{s_{ID}+s_{ID}^{\prime }}$,
    $$\begin{array}{ccc} dk_{ID,t,1} & = & sk_{ID,1}\cdot F_{W,1}{\left(ID\right)}^{r_{ID}^{\prime }}\cdot vk_{ID,t,1}\cdot F_{W,2}{\left(T_{ID}\right)}^{s_{ID}^{\prime }}\\ & = & g_{2}^{\alpha +\beta }\cdot F_{W,1}{\left(ID\right)}^{r_{ID}+r_{ID}^{\prime }}\cdot F_{W,2}{\left(T\right)}^{s_{ID}+s_{ID}^{\prime }}. \end{array}$$
  3. Output a signing key *dk*_*ID*,*t*_ = (*dk*_*ID*,*t*,1_, *dk*_*ID*,*t*,2_, *dk*_*ID*,*t*,3_).
- **ReKey**: On input two signing keys *dk*_*A*,*t*_ = (*dk*_*A*,*t*,1_, *dk*_*A*,*t*,2_, *dk*_*A*,*t*,3_) and *dk*_*B*,*t*_ = (*dk*_*B*,*t*,1_, *dk*_*B*,*t*,2_, *dk*_*B*,*t*,3_) corresponding to two identities *ID*_*A*_ and *ID*_*B*_, the proxy outputs a re-signing key
  $$\begin{array}{ccc} rk_{A\to B,t} & = & \left(rk_{A\to B,t,1},rk_{A\to B,t,2},rk_{A\to B,t,3}\right)\\ & = & (dk_{B,t,1}/dk_{A,t,1},dk_{B,t,2}/dk_{A,t,2},dk_{B,t,3}/dk_{A,t,3}). \end{array}$$
- **Sign**: Given a message *M*, a signer randomly chooses *r*_*m*_ ∈ *Z*_*p*_ and uses the signing key *dk*_*ID*,*t*_ = (*dk*_*ID*,*t*,1_, *dk*_*ID*,*t*,2_, *dk*_*ID*,*t*,3_) to compute $\sigma_{ID,1}=dk_{ID,t,1}\cdot F_{W,3}{\left(M\right)}^{r_{m}}$, *σ*_*ID*,2_ = *dk*_*ID*,*t*,2_, *σ*_*ID*,3_ = *dk*_*ID*,*t*,3_ and $\sigma_{ID,4}=g^{r_{m}}$. Then, the signer outputs a signature *σ*_*ID*_ = (*σ*_*ID*,1_, *σ*_*ID*,2_, *σ*_*ID*,3_, *σ*_*ID*,4_) on *M*.
- **ReSign**: Given a re-signing key *rk*_*A*→*B*,*t*_ = (*rk*_*A*→*B*,*t*,1_, *rk*_*A*→*B*,*t*,2_, *rk*_*A*→*B*,*t*,3_) and a signature *σ*_*A*_ = (*σ*_*A*,1_, *σ*_*A*,2_, *σ*_*A*,3_, *σ*_*A*,4_) on a message *M* with respect to an identity *ID*_*A*_ and a time period *t*, the proxy outputs ⊥ if **Verify**(*pp*,*ID*_*A*_, *t*, *M*, *σ*_*A*_) = 0; otherwise, the proxy randomly chooses $r_{m}^{\prime }\in Z_{p}$, and computes $\sigma_{B,1}=\sigma_{A,1}\cdot rk_{A\to B,t,1}\cdot F_{W,2}{\left(M\right)}^{r_{m}^{\prime }}$, *σ*_*B*,2_ = *σ*_*A*,2_ ⋅ *rk*_*A*→*B*,*t*,2_, *σ*_*B*,3_ = *σ*_*A*,3_ ⋅ *rk*_*A*→*B*,*t*,3_ and $\sigma_{B,4}=\sigma_{A,4}\cdot g^{r_{m}^{\prime }}$. Then, the proxy outputs a signature *σ*_*B*_ = (*σ*_*B*,1_, *σ*_*B*,2_, *σ*_*B*,3_, *σ*_*B*,4_) for *M* with respect to the identity *ID*_*B*_ and the time period *t*.
- **Verify**: Given an identity *ID*, a time period *t* and a signature *σ*_*ID*_ = (*σ*_*ID*,1_, *σ*_*ID*,2_, *σ*_*ID*,3_, *σ*_*ID*,4_) on a message *M*, the verifier first computes *T*_*ID*_ = *H*(*ID*, *t*). Then, the verifier checks whether the following equation holds:
  $$\begin{array}{ccc} e(\sigma_{ID,1},g) & = & e\left(g_{2},g_{1}\right)e\left(F_{W,1}\left(ID\right),\sigma_{ID,2}\right)e\left(F_{W,2}\left(T\right),\sigma_{ID,3}\right)e\left(F_{W,3}\left(M\right),\sigma_{ID,4}\right). \end{array}$$
  If the above equation holds, the verifier accepts that *σ*_*ID*_ is valid and outputs 1; otherwise, the verifier outputs 0.

#### Correctness

For any signature *σ*_*A*_ = (*σ*_*A*,1_, *σ*_*A*,2_, *σ*_*A*,3_, *σ*_*A*,4_) of message *M* on (*ID*_*A*_, *t*), any re-signing key *rk*_*A*→*B*,*t*_ = (*rk*_*A*→*B*,*t*,1_, *rk*_*A*→*B*,*t*,2_, *rk*_*A*→*B*,*t*,3_) = (*dk*_*B*,*t*,1_/*dk*_*A*,*t*,1_, *dk*_*B*,*t*,2_/*dk*_*A*,*t*,2_, *dk*_*B*,*t*,3_/*dk*_*A*,*t*,3_) and *ID*_*B*_’s signing key *dk*_*B*,*t*_ = (*dk*_*B*,*t*,1_, *dk*_*B*,*t*,2_, *dk*_*B*,*t*,3_), where $dk_{B,t,1}=g_{2}^{\alpha +\beta }F_{W,1}{\left(ID_{B}\right)}^{r_{B}+r_{B}^{\prime }}F_{W,2}{\left(T_{B}\right)}^{s_{B}+s_{B}^{\prime }}$, $dk_{B,t,2}=g^{r_{B}+r_{B}^{\prime }}$ and $dk_{B,t,3}=g^{s_{B}+s_{B}^{\prime }}$. Then, we have a re-signature *σ*_*B*_ = (*σ*_*B*,1_, *σ*_*B*,2_, *σ*_*B*,3_, *σ*_*B*,4_) of *M* with respect to identity *ID*_*B*_ and time period *t*, where

$$\begin{array}{ccc} \sigma_{B,1} & = & \sigma_{A,1}\cdot rk_{A\to B,t,1}\cdot F_{W,2}{\left(M\right)}^{r_{m}^{\prime }}\\ & = & dk_{A,t,1}\cdot F_{W,3}{\left(M\right)}^{r_{m}}\cdot \left(dk_{B,t,1}/dk_{A,t,1}\right)\cdot F_{W,2}{\left(M\right)}^{r_{m}^{\prime }}\\ & = & dk_{B,t,1}\cdot F_{W,3}{\left(M\right)}^{r_{m}+r_{m}^{\prime }}\\ & = & g_{2}^{\alpha +\beta }F_{W,1}{\left(ID_{B}\right)}^{r_{B}+r_{B}^{\prime }}F_{W,2}{\left(T_{B}\right)}^{s_{B}+s_{B}^{\prime }}F_{W,3}{\left(M\right)}^{r_{m}+r_{m}^{\prime }}, \end{array}$$

$$\begin{array}{ccc} \sigma_{B,2} & = & \sigma_{A,2}\cdot rk_{A\to B,t,2}=dk_{A,t,2}\cdot \left(dk_{B,t,2}/dk_{A,t,2}\right)=dk_{B,t,2}=g^{r_{B}+r_{B}^{\prime }},\\ \sigma_{B,3} & = & \sigma_{A,3}rk_{A\to B,t,3}=dk_{A,t,3}\cdot \left(dk_{B,t,3}/dk_{A,t,3}\right)=dk_{B,t,3}=g^{s_{B}+s_{B}^{\prime }},\\ \sigma_{B,4} & = & \sigma_{A,4}\cdot g^{r_{m}^{\prime }}=g^{r_{m}}\cdot g^{r_{m}^{\prime }}={g^{r_{m+}}}^{r_{m}^{\prime }}. \end{array}$$

The following equation shows that our RIDPRS scheme satisfies correctness:

$$\begin{array}{ccc} e(\sigma_{B,1},g) & = & e(g_{2}^{\alpha +\beta }F_{W,1}{\left(ID_{B}\right)}^{r_{B}+r_{B}^{\prime }}F_{W,2}{\left(T_{B}\right)}^{s_{B}+s_{B}^{\prime }}F_{W,3}{\left(M\right)}^{r_{m}+r_{m}^{\prime }},g)\\ & = & e\left(g_{2}^{\alpha +\beta },g\right)e\left(F_{W,1}{\left(ID_{B}\right)}^{r_{B}+r_{B}^{\prime }},g\right)e\left(F_{W,2}{\left(T_{B}\right)}^{s_{B}+s_{B}^{\prime }},g\right)e\left(F_{W,3}{\left(M\right)}^{r_{m}+r_{m}^{\prime }},g\right)\\ & = & e\left(g_{2},g^{\alpha +\beta }\right)e\left(F_{W,1}\left(ID_{B}\right),g^{r_{B}+r_{B}^{\prime }}\right)e\left(F_{W,2}\left(T_{B}\right),g^{s_{B}+s_{B}^{\prime }}\right)e\left(F_{W,3}\left(M\right),g^{r_{m}+r_{m}^{\prime }}\right)\\ & = & e\left(g_{2},g_{1}\right)e\left(F_{W,1}\left(ID_{B}\right),\sigma_{B,2}\right)e\left(F_{W,2}\left(T_{B}\right),\sigma_{B,3}\right)e\left(F_{W,3}\left(M\right),\sigma_{B,4}\right). \end{array}$$

From the above equation, we can see that *σ*_*B*_ satisfies the condition **Verify**(*pp*, *ID*_*B*_, *t*, *M*, *σ*_*B*_) = 1, so our RIDPRS scheme is correct. The distribution of the re-signature *σ*_*B*_ generated by the algorithm **ReSign** is the same as that of the signature generated by *ID*_*B*_ himself using the algorithm **Sign**, which demonstrates that our RIDPRS scheme is multi-use. By using the re-signing key *rk*_*A*→*B*,*t*_ between *ID*_*A*_ and *ID*_*B*_, it is easy to compute the re-signing key *rk*_*B*→*A*,*t*_ = 1/*rk*_*A*→*B*,*t*_ between *ID*_*B*_ and *ID*_*A*_, which implies that our RIDPRS scheme is bidirectional.

#### Security analysis

In our RIDPRS scheme, the algorithm **SKGen** re-randomizes a secret key *sk*_*ID*_ and an update key *vk*_*ID*,*t*_ to generate a corresponding signing key for an identity *ID* and time period *t*. Even if an adversary obtains the signing key *dk*_*ID*,*t*_, it is difficult to extract *ID*’s secret key *sk*_*ID*_ from *dk*_*ID*,*t*_. Moreover, the exposure of *dk*_*ID*,*t*_ at time period *t* does not reveal any information concerning other signing keys for identity *ID* at other time periods. Therefore, our RIDPRS scheme can resist signing key exposure attacks.

To simplify the security analysis of our RIDPRS scheme, we classify adversaries into two types: external and internal. The main difference between these two types of attackers is that an external adversary is not allowed to make queries about the secret key of the challenged identity ID* whereas an internal adversary is not allowed to make queries about the update key of the challenged identity ID* or the challenged time period t*. The following two lemmas prove that our RIDPRS scheme is existentially unforgeable against adaptive chosen identity and message attacks.

**Lemma 1.** If a polynomial-time external adversary $A_{1}$ breaks our RIDPRS scheme with non-negligible probability, then there exists an algorithm B that can solve the CDH problem with non-negligible probability.

**Proof.** Assume that an adversary $A_{1}$ breaks the existential unforgeability of our RIDPRS scheme with the probability *ε* after making at most *q*_*sk*_ secret key queries, *q*_*vk*_ update key queries, *q*_*dk*_ signing key queries, *q*_*rk*_ re-signing key queries and *q*_*s*_ signing queries. We will construct an algorithm B to solve the CDH problem in *G*_1_ with the probability at least *ε*_1_ by using $A_{1}$’s forgery. Given a random instance $\left(g,g^{a},g^{b}\right)\in G_{1}^{3}$ of the CDH problem, the goal of B is to compute *g*^*ab*^. The algorithm B interacts with $A_{1}$ as follows.

**Setup:** B randomly chooses two integers *k*_*u*_(0 ≤ *k*_*u*_ ≤ *n*_*u*_) and *k*_*m*_(0 ≤ *k*_*m*_ ≤ *n*_*m*_), and sets *g*_2_ = *g*^*b*^, *l*_*u*_ = 2(*q*_*sk*_ + *q*_*dk*_ + *q*_*rk*_ + *q*_*s*_) and *l*_*m*_ = 2*q*_*s*_ such that *l*_*u*_(*n*_*u*_ + 1)<*p* and *l*_*m*_(*n*_*m*_ + 1)<*p*. To generate the public parameters *pp*, B executes the following operations:

1. Randomly choose *x*_0_, *x*_1_, …, *x*_*n*_*u*__ ∈ *Z*_*l*_*u*__ and *y*_0_, *y*_1_, …, *y*_*n*_*u*__ ∈ *Z*_*p*_, then compute $u_{0}=g_{2}^{-l_{u}k_{u}+x_{0}}g^{y_{0}}$ and a vector $\vec{u}=\left(u_{i}\right)$, where $u_{i}=g_{2}^{x_{i}}g^{y_{i}}$ for 1 ≤ *i* ≤ *n*_*u*_.
2. Randomly choose *z*_0_, *z*_1_, …, *z*_*n*_*v*__ ∈ *Z*_*p*_ and then compute $v_{0}=g^{z_{0}}$ and a vector $\vec{v}=\left(v_{j}\right)$, where $v_{j}=g^{z_{j}}$ for 1 ≤ *j* ≤ *n*_*v*_.
3. Randomly choose *c*_0_, *c*_1_, …, *c*_*n*_*m*__ ∈ *Z*_*l*_*m*__ and *d*_0_, *d*_1_, …, *y*_*n*_*m*__ ∈ *Z*_*p*_ and then compute $w_{0}=g_{2}^{-l_{m}k_{m}+c_{0}}g^{d_{0}}$ and a vector $\vec{w}=\left(w_{k}\right)$, where $w_{k}=g_{2}^{c_{k}}g^{d_{k}}$ for 1 ≤ *k* ≤ *n*_*m*_.
4. Select a collision-resistant hash function $H:{\left\{0,1\right\}}^{*}\to {\left\{0,1\right\}}^{n_{v}}$.
5. Choose a random integer $\beta \in Z_{p}^{*}$ and compute $g_{1}=g^{a}g^{\beta }=g^{a+\beta }$. This calculation implies that the master secret key is $msk=(g_{2}^{a},g_{2}^{\beta })$ but *a* is unknown to B.
6. Send the public parameters (*G*_1_, *G*_2_, *e*, *p*, *g*, *g*_1_, *g*_2_, *H*, *u*_0_, *v*_0_, $w_{0},\vec{u},\vec{v},\vec{w})$ to $A_{1}$.

For any identity $ID=\left(ID_{1},\ldots ,ID_{n_{u}}\right)\in {\left\{0,1\right\}}^{n_{u}}$, any string $T=\left(T_{1},\ldots ,T_{n_{v}}\right)\in {\left\{0,1\right\}}^{n_{v}}$ and any message $M=\left(M_{1},\ldots ,M_{n_{m}}\right)\in {\left\{0,1\right\}}^{n_{m}}$, we define the following five functions:

$F\left(ID\right)=x_{0}-l_{u}k_{u}+\sum_{i=1}^{n_{u}}x_{i}ID_{i}$, $J\left(ID\right)=y_{0}+\sum_{i=1}^{n_{u}}y_{i}ID_{i}$, $E\left(T\right)=z_{0}+\sum_{j=1}^{n_{v}}z_{j}T_{j}$, $K\left(M\right)=c_{0}-l_{m}k_{m}+\sum_{k=1}^{n_{m}}c_{k}M_{k}$, $L\left(M\right)=d_{0}+\sum_{k=1}^{n_{m}}d_{k}M_{k}$.

Thus, we have

$$\begin{array}{ccc} F_{W,1}\left(ID\right) & = & u_{0}\prod_{i=1}^{n_{u}}{\left(u_{i}\right)}^{ID_{i}}=g_{2}^{F(ID)}g^{J(ID)},\\ F_{W,2}\left(T\right) & = & v_{0}\prod_{j=1}^{n_{v}}{\left(v_{j}\right)}^{T_{j}}=g^{E(T)},\\ F_{W,3}\left(M\right) & = & w_{0}\prod_{k=1}^{n_{m}}{\left(w_{k}\right)}^{M_{k}}=g_{2}^{K(M)}g^{L(M)}. \end{array}$$

**Queries:** The adversary $A_{1}$ can issue the following types of queries adaptively:

- *Extract-query:* When $A_{1}$ asks for a secret key query on an identity *ID*, B first computes *F*(*ID*). We note that F(ID)=0modp implies $F\left(ID\right)=0\;\mathrm{mod}\;\;l_{u}$ (and thus $F\left(ID\right)\neq 0\;\mathrm{mod}\;\;l_{u}$ implies F(ID)≠0modp). If $F\left(ID\right)=0\;\mathrm{mod}\;\;l_{u}$, B aborts; otherwise, B randomly chooses *r*_*ID*_ ∈ *Z*_*p*_ computes
  $$\begin{array}{ccc} sk_{ID} & = & \left(sk_{ID,1},sk_{ID,2}\right)\\ & = & ({\left(g^{a}\right)}^{\frac{-J(ID)}{F(ID)}}F_{W,1}{\left(ID\right)}^{r_{ID}},{\left(g^{a}\right)}^{\frac{-1}{F(ID)}}g^{r_{ID}}), \end{array}$$
  and then sends the secret key *sk*_*ID*_ to $A_{1}$.
- *KeyUp-query:* When $A_{1}$ issues an update key query on an identity *ID* and a time period *t*, B randomly chooses *s*_*ID*_ ∈ *Z*_*p*_ and computes *T*_*ID*_ = *H*(*ID*, *t*). Then, B uses the secret value *β* to compute
  $$\begin{array}{c} vk_{ID,t}=\left(vk_{ID,t,1},vk_{ID,t,2}\right)=\left(g_{2}^{\beta }F_{W,2}{\left(T_{ID}\right)}^{s_{ID}},g^{s_{ID}}\right) \end{array}$$
  and returns an update key *vk*_*ID*,*t*_ to $A_{1}$.
- *SKGen-query:* For a signing key query on (*ID*, *t*), if identity *ID* has been revoked at time period *t*, B outputs ⊥. Otherwise, B randomly chooses $r_{ID}^{\prime },s_{ID}^{\prime }\in Z_{p}$, and performs an *Extract-query* on *ID* and a *KeyUp-query* on (*ID*, *t*) to obtain a secret key *sk*_*ID*_ = (*sk*_*ID*,1_, *sk*_*ID*,2_) and an update key *vk*_*ID*,*t*_ = (*vk*_*ID*,*t*,1_, *vk*_*ID*,*t*,2_), respectively. Then, B computes *T*_*ID*_ = *H*(*ID*, *t*),
  $$\begin{array}{ccc} dk_{ID,t,1} & = & sk_{ID,1}F_{W,1}{\left(ID\right)}^{r_{ID}^{\prime }}\cdot vk_{ID,t,1}F_{W,2}{\left(T_{ID}\right)}^{s_{ID}^{\prime }},\\ dk_{ID,t,2} & = & sk_{ID,2}\cdot g^{r_{ID}^{\prime }},\;dk_{ID,t,3}=vk_{ID,t,2}\cdot g^{s_{ID}^{\prime }}, \end{array}$$
  and returns a signing key *dk*_*ID*,*t*_ = (*dk*_*ID*,*t*,1_, *dk*_*ID*,*t*,2_, *dk*_*ID*,*t*,3_) to $A_{1}$.
- *ReKey-query:* When $A_{1}$ issues a re-signing key query on two identities (*ID*_*A*_, *ID*_*B*_) and a time period *t*, B first makes *SKGen-query* on (*ID*_*A*_, *t*) and (*ID*_*B*_, *t*) to obtain the corresponding signing keys *dk*_*A*,*t*_ and *dk*_*B*,*t*_, respectively. Then, B executes the algorithm **ReKey**(*pp*, *dk*_*A*,*t*_, *dk*_*B*,*t*_) to produce a re-signing key *rk*_*A*→*B*,*t*_ and sends it to $A_{1}$.
- *Sign-query:* When $A_{1}$ issues a signature query on a message *M* with respect to an identity *ID* and a time period *t*, B considers the following two cases:
  – Case 1: If $F\left(ID\right)\neq 0\;\mathrm{mod}\;\;l_{u}$, B makes a *SKGen-query* on (*ID*, *t*) to obtain a signing key *dk*_*ID*,*t*_. Then, B executes the algorithm **Sign**(*pp*, *dk*_*ID*,*t*_, *M*) to produce a signature *σ* on *M* and returns it to $A_{1}$.
  – Case 2: If $F\left(ID\right)=0\;\mathrm{mod}\;\;l_{u}$, B further considers the following two subcases:
    * Case 2.1: If $K\left(M\right)=0\;\mathrm{mod}\;\;l_{m}$, B aborts.
    * Case 2.2: If $K\left(M\right)\neq 0\;\mathrm{mod}\;\;l_{m}$, B randomly chooses ${\tilde{r}}_{ID},{\tilde{s}}_{ID},{\tilde{r}}_{m}\in Z_{p}$, and computes *T*_*ID*_ = *H*(*ID*, *t*), $\sigma_{2}=g^{{\tilde{r}}_{ID}}$, $\sigma_{3}=g^{{\tilde{s}}_{ID}}$, $\sigma_{4}={\left(g^{a}\right)}^{-1/K(M)}g^{{\tilde{r}}_{m}}$ and
      $$\begin{array}{c} \sigma_{1}=g_{2}^{\beta }F_{W,1}{\left(ID\right)}^{{\tilde{r}}_{ID}}F_{W,2}{\left(T_{ID}\right)}^{{\tilde{s}}_{ID}}{\left(g^{a}\right)}^{{}^{\frac{-L(M)}{K(M)}}}F_{W,3}{\left(M\right)}^{{\tilde{r}}_{m}}. \end{array}$$
      Then, B returns a signature *σ* = (*σ*_1_, *σ*_2_, *σ*_3_, *σ*_4_) on *M* to $A_{1}$.

**Forgery:** The adversary $A_{1}$ finally outputs a signature $\sigma^{*}=\left(\sigma_{1}^{*},\sigma_{2}^{*},\sigma_{3}^{*},\sigma_{4}^{*}\right)$ on a message M* with respect to an identity ID* and a time period t*. If $F(ID^{*})\neq 0\;\mathrm{mod}\;\;p$ or $K(M^{*})\neq 0\;\mathrm{mod}\;\;p$, B aborts. Otherwise, to solve the instance of the CDH problem, B computes *T*_ID*_ = *H*(ID*, t*) and outputs *g*^*ab*^ as follows:

$$\begin{array}{ccc} & & \frac{\sigma_{1}^{*}}{g_{2}^{\beta }{\left(\sigma_{2}^{*}\right)}^{J(ID^{*})}{\left(\sigma_{3}^{*}\right)}^{E(T_{ID^{*}})}{\left(\sigma_{4}^{*}\right)}^{L(M^{*})}}\\ & & \;=\frac{g_{2}^{a+\beta }F_{W,1}{\left(ID^{*}\right)}^{{\tilde{r}}_{ID^{*}}}F_{W,2}{\left(T_{ID^{*}}\right)}^{{\tilde{s}}_{ID^{*}}}F_{W,3}{\left(M^{*}\right)}^{{\tilde{r}}_{m^{*}}}}{g_{2}^{\beta }{\left(g^{{\tilde{r}}_{ID^{*}}}\right)}^{J(ID^{*})}{\left(g^{{\tilde{s}}_{ID^{*}}}\right)}^{E(T_{ID^{*}})}{\left(g^{{\tilde{r}}_{m^{*}}}\right)}^{L(M^{*})}}\\ & & \;=\frac{g_{2}^{a}{\left(g_{2}^{F(ID^{*})}g^{J(ID^{*})}\right)}^{{\tilde{r}}_{ID^{*}}}{\left(g^{E(T_{ID^{*}})}\right)}^{{\tilde{s}}_{ID^{*}}}{\left(g_{2}^{K(M^{*})}g^{L(M^{*})}\right)}^{{\tilde{r}}_{m^{*}}}}{{\left(g^{J(ID^{*})}\right)}^{{\tilde{r}}_{ID^{*}}}{\left(g^{E(T_{ID^{*}})}\right)}^{{\tilde{s}}_{ID^{*}}}{\left(g^{L(M^{*})}\right)}^{{\tilde{r}}_{m^{*}}}}\\ & & \;=g_{2}^{a}\;\left(\text{since}\;F\left(ID^{*}\right)=K\left(M^{*}\right)=0\;\mathrm{mod}\;\;p\right)\\ & & \;=g^{ab}. \end{array}$$

Now, we analyse the probability that B does not abort in the above simulation. If B completes the simulation without aborting, then the following events must have occurred:

- In the *Extract-query*, *SKGen-query* and *ReKey-query* phases, the condition $F\left(ID\right)\neq 0\;\mathrm{mod}\;\;l_{u}$ must be satisfied for any queried identity *ID*.
- In the *Sign-query* phase, $F\left(ID\right)\neq 0\;\mathrm{mod}\;\;l_{u}$ or $K\left(M\right)\neq 0\;\mathrm{mod}\;\;l_{m}$ must be satisfied for any queried identity *ID* and message *M*.
- In the forgery phase, $F(ID^{*})=0\;\mathrm{mod}\;\;p$ and $K(M^{*})=0\;\mathrm{mod}\;\;p$ must be satisfied for the challenged identity ID* and message M*.

Similar to the probability analysis described in [20, 33], we define the following events:

- $X_{i}:F\left(ID_{i}\right)\neq 0\;\mathrm{mod}\;l_{u}$ for *i* = 1, …, *q*_*sk*_ + *q*_*dk*_ + *q*_*rk*_ + *q*_*s*_.
- $X^{*}:F\left(ID^{*}\right)=0\;\mathrm{mod}\;p$.
- $Y_{j}:K\left(M_{j}\right)\neq 0\;\mathrm{mod}\;l_{m}$ for *i* = 1, …, *q*_*s*_.
- $Y^{*}:K\left(M^{*}\right)=0\;\mathrm{mod}\;p$.

Hence, the probability that B will not abort in the above simulation is

$$\begin{array}{ccc} \mathrm{Pr}[\neg abort] & = & \mathrm{Pr}[\overset{q_{sk}+q_{dk}+q_{rk}+q_{s}}{\underset{i=1}{⋂}}X_{i}\cap X^{*}\cap \overset{q_{s}}{\underset{j=1}{⋂}}Y_{j}\cap Y^{*}]\\ & \geq & \mathrm{Pr}\left[\overset{q_{sk}+q_{dk}+q_{rk}+q_{s}}{\underset{i=1}{⋂}}X_{i}\cap X^{*}\right]\cdot \mathrm{Pr}\left[\overset{q_{s}}{\underset{j=1}{⋂}}Y_{j}\cap Y^{*}\right]\\ & = & \mathrm{Pr}\left[X^{*}\right]\cdot \mathrm{Pr}\left[\overset{q_{sk}+q_{dk}+q_{rk}+q_{s}}{\underset{i=1}{⋂}}X_{i}|X^{*}\right]\cdot \mathrm{Pr}\left[Y^{*}\right]\cdot \mathrm{Pr}\left[\overset{q_{s}}{\underset{j=1}{⋂}}Y_{j}|Y^{*}\right]. \end{array}$$

Because *l*_*u*_(*n*_*u*_ + 1) < *p*, we can find that

$$\begin{array}{ccc} \mathrm{Pr}[X^{*}] & = & \mathrm{Pr}[F\left(ID^{*}\right)=0\;\mathrm{mod}\;p]\\ & \geq & \mathrm{Pr}[F\left(ID^{*}\right)=0\;\mathrm{mod}\;p\cap F\left(ID^{*}\right)=0\;\mathrm{mod}\;l_{u}]\\ & = & \mathrm{Pr}\left[F\left(ID^{*}\right)=0\;\mathrm{mod}\;l_{u}\right]\mathrm{Pr}\left[F\left(ID^{*}\right)=0\;\mathrm{mod}\;p|F\left(ID^{*}\right)=0\;\mathrm{mod}\;l_{u}\right]\\ & = & \frac{1}{l_{u}}\frac{1}{n_{u}+1}, \end{array}$$

and

$$\begin{array}{ccc} \mathrm{Pr}[\overset{q_{sk}+q_{dk}+q_{rk}+q_{s}}{\underset{i=1}{⋂}}X_{i}|X^{*}] & = & 1-\mathrm{Pr}[\overset{q_{sk}+q_{dk}+q_{rk}+q_{s}}{\underset{i=1}{⋃}}\neg X_{i}|X^{*}]\\ & \geq & 1-\sum_{i=1}^{q_{sk}+q_{dk}+q_{rk}+q_{s}}\mathrm{Pr}[\neg X_{i}|X^{*}]\\ & = & 1-\frac{q_{sk}+q_{dk}+q_{rk}+q_{s}}{l_{u}}. \end{array}$$

Since *l*_*m*_(*n*_*m*_ + 1) < *p*, we have that

$$\begin{array}{ccc} \mathrm{Pr}[Y^{*}] & = & \mathrm{Pr}[K\left(M^{*}\right)=\;\mathrm{mod}\;\;p]\\ & \geq & \mathrm{Pr}[K\left(M^{*}\right)=\;\mathrm{mod}\;\;p\cap K\left(M^{*}\right)=\;\mathrm{mod}\;\;l_{m}]\\ & = & \mathrm{Pr}\left[K\left(M^{*}\right)=\;\mathrm{mod}\;\;l_{m}\right]\mathrm{Pr}\left[K\left(M^{*}\right)=\;\mathrm{mod}\;\;p|K\left(M^{*}\right)=\;\mathrm{mod}\;\;l_{m}\right]\\ & = & \frac{1}{l_{m}}\frac{1}{n_{m}+1},\\ \mathrm{Pr}[\overset{q_{s}}{\underset{j=1}{⋂}}Y_{j}|Y^{*}] & = & 1-\mathrm{Pr}[\overset{q_{s}}{\underset{j=1}{⋃}}\neg Y_{j}|Y^{*}]\\ & \geq & 1-\sum_{j=1}^{q_{s}}\mathrm{Pr}[\neg Y_{j}|Y^{*}]\\ & = & 1-\frac{q_{s}}{l_{m}}. \end{array}$$

As *l*_*u*_ = 2(*q*_*sk*_ + *q*_*dk*_ + *q*_*rk*_ + *q*_*s*_) and *l*_*m*_ = 2*q*_*s*_, we can obtain the resulting probability

$$\begin{array}{ccc} \mathrm{Pr}[\neg abort] & = & \frac{1}{l_{u}}\frac{1}{n_{u}+1}\left(1-\frac{q_{sk}+q_{dk}+d_{rk}+q_{s}}{l_{u}}\right)\frac{1}{l_{m}}\frac{1}{n_{m}+1}\left(1-\frac{q_{s}}{l_{m}}\right)\\ & = & \frac{1}{2(q_{sk}+q_{dk}+d_{rk}+q_{s})}\frac{1}{n_{u}+1}\left(1-\frac{q_{sk}+q_{dk}+d_{rk}+q_{s}}{2(q_{sk}+q_{dk}+d_{rk}+q_{s})}\right)\\ & \cdot & \frac{1}{2q_{s}}\frac{1}{n_{m}+1}\left(1-\frac{q_{s}}{2q_{s}}\right)\\ & = & \frac{1}{16\left(n_{u}+1\right)\left(n_{m}+1\right)q_{s}\left(q_{sk}+q_{dk}+d_{rk}+q_{s}\right)}. \end{array}$$

Therefore, B can successfully solve the CDH problem in *G*_1_ with a probability of $\varepsilon_{1}>\frac{\varepsilon }{16\left(n_{u}+1\right)\left(n_{m}+1\right)q_{s}\left(q_{sk}+q_{dk}+q_{rk}+q_{s}\right)}$.

**Lemma 2.** If a polynomial-time internal adversary $A_{2}$ breaks our RIDPRS scheme with a non-negligible probability, then there exists an algorithm B that can solve the CDH problem with non-negligible probability.

**Proof.** Assume that an adversary $A_{2}$ breaks the existential unforgeability of our RIDPRS scheme with the probability *ε* after making at most *q*_*sk*_ secret key queries, *q*_*vk*_ update key queries, *q*_*dk*_ signing key queries, *q*_*rk*_ re-signing key queries and *q*_*s*_ signing queries. We can construct an algorithm B that solves the CDH problem in *G*_1_ with a probability *ε*_2_ using the adversary’s forgery $A_{2}$. B is given a random instance $\left(g,g^{a},g^{b}\right)\in G_{1}^{3}$ of the CDH problem. To calculate *g*^*ab*^, B interacts with $A_{2}$ as follows.

**Setup:** B randomly chooses two integers *k*_*v*_(0 ≤ *k*_*v*_ ≤ *n*_*v*_) and *k*_*m*_(0 ≤ *k*_*m*_ ≤ *n*_*m*_), and sets *g*_2_ = *g*^*b*^, *l*_*v*_ = 2(*q*_*vk*_ + *q*_*dk*_ + *q*_*rk*_ + *q*_*s*_) and *l*_*m*_ = 2*q*_*s*_ such that *l*_*v*_(*n*_*v*_ + 1) < *p* and *l*_*m*_(*n*_*m*_ + 1) < *p*. Then, B generate public parameters *pp* according to the following steps:

1. Randomly choose *x*_0_, *x*_1_, …, *x*_*n*_*v*__ ∈ *Z*_*l*_*v*__ and *y*_0_, *y*_1_, …, *y*_*n*_*v*__ ∈ *Z*_*p*_; then, compute $v_{0}=g_{2}^{-l_{v}k_{v}+x_{0}}g^{y_{0}}$ and a vector $\vec{v}=\left(v_{j}\right)$, where $v_{j}=g_{2}^{x_{j}}g^{y_{j}}$ for 1 ≤ *j* ≤ *n*_*v*_.
2. Randomly choose *z*_0_, *z*_1_, …, *z*_*n*_*u*__ ∈ *Z*_*p*_; then, compute $u_{0}=g^{z_{0}}$ and a vector $\vec{u}=\left(u_{i}\right)$, where $u_{i}=g^{z_{i}}$ for 1 ≤ *i* ≤ *n*_*u*_.
3. Set parameter *w*_0_ and vector $\vec{w}=\left(w_{k}\right)$ in the same way as is done in Lemma 1.
4. Select a collision-resistant hash function $H:{\left\{0,1\right\}}^{*}\to {\left\{0,1\right\}}^{n_{v}}$.
5. Choose a random integer $\tau \in Z_{p}^{*}$ and compute $g_{1}=g^{a}g^{\tau }=g^{a+\tau }$, which implies that the master secret key is $msk=(g_{2}^{\tau },g_{2}^{a})$, but *a* is unknown to B.
6. Send the public parameters (*G*_1_, *G*_2_, *e*, *p*, *g*, *g*_1_, *g*_2_, *H*, *u*_0_, *v*_0_, $w_{0},\vec{u},\vec{v},\vec{w})$ to $A_{2}$.

For an identity $ID=\left(ID_{1},\ldots ,ID_{n_{u}}\right)\in {\left\{0,1\right\}}^{n_{u}}$, a string $T=\left(T_{1},\ldots ,T_{n_{v}}\right)\in {\left\{0,1\right\}}^{n_{v}}$ and a message $M=\left(M_{1},\ldots ,M_{n_{m}}\right)\in {\left\{0,1\right\}}^{n_{m}}$, we define five functions:

$E_{2}\left(ID\right)=z_{0}+\sum_{i=1}^{n_{u}}z_{i}ID_{i}$, $F_{2}\left(T\right)=x_{0}-l_{v}k_{v}+\sum_{i=1}^{n_{v}}x_{i}T_{i}$, $J_{2}\left(T\right)=y_{0}+\sum_{i=1}^{n_{v}}y_{i}T_{i}$, $K\left(M\right)=c_{0}-l_{m}k_{m}+\sum_{k=1}^{n_{m}}c_{k}M_{k}$, $L\left(M\right)=d_{0}+\sum_{k=1}^{n_{m}}d_{k}M_{k}$.

Thus, we also have

$$\begin{array}{ccc} F_{W,1}\left(ID\right) & = & u_{0}\prod_{i=1}^{n_{u}}{\left(u_{i}\right)}^{ID_{i}}=g^{E_{2}\left(ID\right)},\\ F_{W,2}\left(T\right) & = & v_{0}\prod_{j=1}^{n_{v}}{\left(v_{j}\right)}^{T_{j}}=g_{2}^{F_{2}\left(T\right)}g^{J_{2}\left(T\right)},\\ F_{W,3}\left(M\right) & = & w_{0}\prod_{k=1}^{n_{m}}{\left(w_{k}\right)}^{M_{k}}=g_{2}^{K(M)}g^{L(M)}. \end{array}$$

**Queries:** B answers $A_{2}$’s queries as follows.

- *Extract-query:* Upon receiving a secret key query for identity *ID*, B randomly chooses *r*_*ID*_ ∈ *Z*_*p*_ and computes
  $$\begin{array}{c} sk_{ID}=\left(sk_{ID,1},sk_{ID,2}\right)=\left(g_{2}^{\tau }F_{W,1}{\left(ID\right)}^{r_{ID}},g^{r_{ID}}\right). \end{array}$$
  Then, B sends a secret key *sk*_*ID*_ to $A_{2}$.
- *KeyUp-query:* Upon receiving an update key query on an identity *ID* and a time period *t*, B first computes *T*_*ID*_ = *H*(*ID*, *t*). If $F_{2}\left(T_{ID}\right)=0\;\mathrm{mod}\;\;l_{v}$, B aborts. Otherwise, B randomly chooses *s*_*ID*_ ∈ *Z*_*p*_ and outputs an update key *vk*_*ID*,*t*_ = (*vk*_*ID*,*t*,1_, *vk*_*ID*,*t*,2_) in response, where
  $$\begin{array}{ccc} vk_{ID,t,1} & = & {\left(g^{a}\right)}^{\frac{-J_{2}\left(T_{ID}\right)}{F_{2}\left(T_{ID}\right)}}F_{W,2}{\left(T_{ID}\right)}^{s_{ID}},\\ vk_{ID,t,2} & = & {\left(g^{a}\right)}^{\frac{-1}{F_{2}\left(T_{ID}\right)}}g^{s_{ID}}. \end{array}$$
- *SKGen-query:* B answers a signing key query in the same way as it does the *SKGen-query* in Lemma 1.
- *ReKey-query:* B answers a re-signing key query in the same way as it does the *ReKey-query* in Lemma 1.
- *Sign-query:* Upon receiving a signature query on a message *M* with respect to an identity *ID* and a time period *t*, B computes *T*_*ID*_ = *H*(*ID*, *t*) and considers the following two cases:
  – Case 1: If $F_{2}\left(T_{ID}\right)\neq 0\;\mathrm{mod}\;\;l_{v}$, B generates a signature on *M* in the same manner as in Case 1 in Lemma 1.
  – Case 2: If $F_{2}\left(T_{ID}\right)=0\;\mathrm{mod}\;\;l_{v}$, B further considers the following two subcases:
    * Case 2.1: If $K\left(M\right)=0\;\mathrm{mod}\;\;l_{m}$, B aborts.
    * Case 2.2: If $K\left(M\right)\neq 0\;\mathrm{mod}\;\;l_{m}$, B creates a signature on *M* in the same manner as in Case 2.2 in Lemma 1, except that
      $$\begin{array}{ccc} \sigma_{1} & = & g_{2}^{\tau }F_{W,1}{\left(ID\right)}^{{\tilde{r}}_{ID}}F_{W,2}{\left(T_{ID}\right)}^{{\tilde{s}}_{ID}}{\left(g^{a}\right)}^{\frac{-L(M)}{K(M)}}F_{W,3}{\left(M\right)}^{{\tilde{r}}_{m}}. \end{array}$$

**Forgery:** The adversary $A_{2}$ finally outputs a signature $\sigma^{*}=\left(\sigma_{1}^{*},\sigma_{2}^{*},\sigma_{3}^{*},\sigma_{4}^{*}\right)$ on a message M* with respect to an identity ID* and a time period t*. B first computes *T*_ID*_ = *H*(ID*, t*). If $F_{2}\left(T_{ID^{*}}\right)\neq 0\;\mathrm{mod}\;\;p$ or $K(M^{*})\neq 0\;\mathrm{mod}\;\;p$, B aborts. Otherwise, B computes *g*^*ab*^ as follows:

$$\begin{array}{ccc} & & \frac{\sigma_{1}^{*}}{g_{2}^{\tau }{\left(\sigma_{2}^{*}\right)}^{E_{2}\left(ID^{*}\right)}{\left(\sigma_{3}^{*}\right)}^{J_{2}\left(T_{ID^{*}}\right)}{\left(\sigma_{4}^{*}\right)}^{L(M^{*})}}\\ & & \;=\frac{g_{2}^{a+\tau }F_{W,1}{\left(ID^{*}\right)}^{{\tilde{r}}_{ID^{*}}}F_{W,2}{\left(T_{ID^{*}}\right)}^{{\tilde{s}}_{ID^{*}}}F_{W,3}{\left(M^{*}\right)}^{{\tilde{r}}_{m^{*}}}}{g_{2}^{\tau }{\left(g^{{\tilde{r}}_{ID^{*}}}\right)}^{E_{2}\left(ID^{*}\right)}{\left(g^{{\tilde{s}}_{ID^{*}}}\right)}^{J_{2}\left(T_{ID^{*}}\right)}{\left(g^{{\tilde{r}}_{m^{*}}}\right)}^{L(M^{*})}}\\ & & \;=\frac{g_{2}^{a}{\left(g^{E_{2}\left(ID^{*}\right)}\right)}^{{\tilde{r}}_{ID^{*}}}{\left(g_{2}^{F_{2}\left(T_{ID^{*}}\right)}g^{J_{2}\left(T_{ID^{*}}\right)}\right)}^{{\tilde{s}}_{ID^{*}}}{\left(g_{2}^{K(M^{*})}g^{L(M^{*})}\right)}^{{\tilde{r}}_{m^{*}}}}{{\left(g^{E_{2}\left(ID^{*}\right)}\right)}^{{\tilde{r}}_{ID^{*}}}{\left(g^{J_{2}\left(T_{ID^{*}}\right)}\right)}^{{\tilde{s}}_{ID^{*}}}{\left(g^{L(M^{*})}\right)}^{{\tilde{r}}_{m^{*}}}}\\ & & \;=g_{2}^{a}\;\left(\text{since}\;F_{2}\left(T_{ID^{*}}\right)=K\left(M^{*}\right)=0\;\mathrm{mod}\;\;p\right)\\ & & \;=g^{ab}. \end{array}$$

Similar to the probabilistic analysis for Lemma 1, the probability that algorithm B successfully solves the CDH problem in *G*_1_ is at least $\frac{\varepsilon }{16\left(n_{v}+1\right)\left(n_{m}+1\right)q_{s}\left(q_{vk}+q_{dk}+q_{rk}+q_{s}\right)}$.

Combining Lemma 1 and Lemma 2, we obtain the following theorem.

**Theorem 1.** Our RIDPRS scheme is existentially unforgeable against adaptive chosen identity and message attacks in the standard model under the CDH assumption.

### Server-aided revocable identity-based proxy re-signature scheme

#### Description

Based on our RIDPRS scheme, we construct a bidirectional SA-RIDPRS scheme in which the verifier can verify the legality of a signature at relatively low computational cost with the help of a server. Our SA-RIDPRS scheme is described as follows:

- The **Setup**, **Extract**, **KeyUp**, **SKGen**, **ReKey**, **Sign**, **ReSign** and **Verify** algorithms are the same as those in our RIDPRS scheme described in Section 4.1.1.
- **SA-Setup:** Given the public parameters *pp*, the verifier chooses a random integer $x\in Z_{p}^{*}$, computes *K*_0_ = *e*(*g*_2_, *g*_1_)^*x*^ and sets a string $VS\text{tring}=(x,K_{0})$.
- **SA-Verify:** Given a signature *σ* = (*σ*_1_, *σ*_2_, *σ*_3_, *σ*_4_) on a message *M* with respect to an identity *ID* and a time period *t*, the verifier interacts with a server through the following protocol:
  1. The verifier computes $\sigma^{\prime }=\left(\sigma_{1}^{\prime },\sigma_{2}^{\prime },\sigma_{3}^{\prime },\sigma_{4}^{\prime }\right)=\left({\left(\sigma_{1}\right)}^{x},{\left(\sigma_{2}\right)}^{x},{\left(\sigma_{3}\right)}^{x},{\left(\sigma_{4}\right)}^{x}\right)$ and sends the tuple $\left(ID,t,M,\sigma_{2}^{\prime },\sigma_{3}^{\prime },\sigma_{4}^{\prime }\right)$ to the server.
  2. The server computes *T*_*ID*_ = *H*(*ID*, *t*), $K_{2}=e\left(F_{W,1}\left(ID\right),\sigma_{2}^{\prime }\right)$, $K_{3}=e\left(F_{W,2}\left(T_{ID}\right),\sigma_{3}^{\prime }\right)$ and $K_{4}=e\left(F_{W,3}\left(M\right),\sigma_{4}^{\prime }\right)$. Then, the server sends (*K*_2_, *K*_3_, *K*_4_) to the verifier.
  3. The verifier first computes $K_{1}=e\left(\sigma_{1}^{\prime },g\right)$ and checks whether the equation *K*_1_ = *K*_0_ ⋅ *K*_2_ ⋅ *K*_3_ ⋅ *K*_4_ holds. If the equation holds, the verifier accepts that *σ* is valid and outputs 1; otherwise, the verifier outputs 0.

#### Correctness

For any signature *σ* = (*σ*_1_, *σ*_2_, *σ*_3_, *σ*_4_) $=(g_{2}^{\alpha +\beta }F_{W,1}{\left(ID\right)}^{{\tilde{r}}_{ID}}F_{W,2}{\left(T_{ID}\right)}^{{\tilde{s}}_{ID}}F_{W,3}{\left(M\right)}^{{\tilde{r}}_{m}}$, $g^{{\tilde{r}}_{ID}},g^{{\tilde{s}}_{ID}},g^{{\tilde{r}}_{m}})$ and a string $VS\text{tring}=(x,K_{0}=e{\left(g_{2},g_{1}\right)}^{x})$, we have

$$\begin{array}{ccc} K_{1} & = & e\left(\sigma_{1}^{\prime },g\right)=e\left({\left(\sigma_{1}\right)}^{x},g\right)\\ & = & e({\left(g_{2}^{\alpha +\beta }F_{W,1}{\left(ID\right)}^{{\tilde{r}}_{ID}}F_{W,2}{\left(T_{ID}\right)}^{{\tilde{s}}_{ID}}F_{W,3}{\left(M\right)}^{{\tilde{r}}_{m}}\right)}^{x},g)\\ & = & e{\left(g_{2},g^{\alpha +\beta }\right)}^{x}e\left(F_{W,1}\left(ID\right),{\left(g^{{\tilde{r}}_{ID}}\right)}^{x}\right)e\left(F_{W,2}\left(T_{ID}\right),{\left(g^{{\tilde{s}}_{ID}}\right)}^{x}\right)e\left(F_{W,3}\left(M\right),{\left(g^{{\tilde{r}}_{m}}\right)}^{x}\right)\\ & = & e{\left(g_{2},g_{1}\right)}^{x}e\left(F_{W,1}\left(ID\right),{\left(\sigma_{2}\right)}^{x}\right)e\left(F_{W,2}\left(T_{ID}\right),{\left(\sigma_{3}\right)}^{x}\right)e\left(F_{W,3}\left(M\right),{\left(\sigma_{4}\right)}^{x}\right)\\ & = & e{\left(g_{2},g_{1}\right)}^{x}e\left(F_{W,1}\left(ID\right),\sigma_{2}^{\prime }\right)e\left(F_{W,2}\left(T_{ID}\right),\sigma_{3}^{\prime }\right)e\left(F_{W,3}\left(M\right),\sigma_{4}^{\prime }\right)\\ & = & K_{0}\cdot K_{2}\cdot K_{3}\cdot K_{4}. \end{array}$$

Thus, the above equation shows that our SA-RIDPRS scheme satisfies the correctness.

#### Security analysis

In Section 4.1.3, we demonstrated that our RIDPRS scheme is existentially unforgeable in the standard model. According to Definition 4, our SA-RIDPRS scheme is secure if we prove that the algorithm **SA-Verify** has the soundness property.

**Lemma 3.** The algorithm **SA-Verify** in our SA-RIDPRS scheme has soundness against adaptive chosen message and collusion attacks.

**Proof.** Assume that the adversary A is acting as a server with powerful computational capabilities, and the challenger C is acting as a verifier. Because A is allowed to collude with the signer or the proxy, A has access to the signing or re-signing key to generate a valid signature for any message. First, A sends an invalid message-signature pair (M*, σ*) to C. Then, A’s task is to convince C that σ* is a valid signature on a message M* with respect to an identity ID* and a time period t*.

**Setup:** C first runs the algorithm **Setup** to generate public parameters *pp*, and runs the **Extract**, **KeyUp** and **SKGen** algorithms to generate a signing key $dk_{ID^{*},t^{*}}^{*}$ with respect to the tuple (ID*, t*). Then, C randomly chooses $x^{*}\in Z_{p}^{*}$, computes $K_{0}^{*}=e{\left(g_{2},g_{1}\right)}^{x^{*}}$ and secretly stores the string $VS\text{tring}=(x^{*},K_{0}^{*})$. Finally, C sends $\left(pp,dk_{ID^{*},t^{*}}^{*},ID^{*},t^{*}\right)$ to the adversary A.

**Queries:** A can adaptively make at most *q*_*sv*_ sever-aided verification queries to C. For each query on the tuple (*ID*_*i*_, *t*_*i*_, *M*_*i*_, *σ*_*i*_), C runs the algorithm **SA-Verify** with A and C returns the resulting output to A.

**Forgery:** The adversary A eventually sends a message-signature pair (M*, σ* = $\left(\sigma_{1}^{*},\sigma_{2}^{*},\sigma_{3}^{*},\sigma_{4}^{*})\right)$ to C, where σ* is not a valid signature on message M* with respect to identity ID* and time period t*, which means that **Verify**(*pp*, ID*, t*, M*, σ*) = 0. Subsequently, C interacts with A as follows.

1. C uses the secret string $VS\text{tring}=(x^{*},K_{0}^{*})$ to compute
  $$\begin{array}{ccc} {\left(\sigma^{*}\right)}^{\prime } & = & \left({\left(\sigma_{1}^{*}\right)}^{\prime },{\left(\sigma_{2}^{*}\right)}^{\prime },{\left(\sigma_{3}^{*}\right)}^{\prime },{\left(\sigma_{4}^{*}\right)}^{\prime }\right)=\left({\left(\sigma_{1}^{*}\right)}^{x^{*}},{\left(\sigma_{2}^{*}\right)}^{x^{*}},{\left(\sigma_{3}^{*}\right)}^{x^{*}},{\left(\sigma_{4}^{*}\right)}^{x^{*}}\right). \end{array}$$
  Then, C sends $\left(ID^{*},t^{*},M^{*},{\left(\sigma_{2}^{*}\right)}^{\prime },{\left(\sigma_{3}^{*}\right)}^{\prime },{\left(\sigma_{4}^{*}\right)}^{\prime }\right)$ to A.
2. A computes *T*_ID*_ = *H*(ID*, t*), $K_{2}^{*}=e(F_{W,1}\left(ID^{*}\right)$, ${\left(\sigma_{2}^{*}\right)}^{\prime })$, $K_{3}^{*}=e\left(F_{W,2}\left(T_{ID^{*}}\right),{\left(\sigma_{3}^{*}\right)}^{\prime }\right)$ and $K_{4}^{*}=e(F_{W,3}\left(M^{*}\right)$, ${\left(\sigma_{4}^{*}\right)}^{\prime })$ and returns $\left(K_{2}^{*},K_{3}^{*},K_{4}^{*}\right)$ to C.
3. C computes $K_{1}^{*}=e\left({\left(\sigma_{1}^{*}\right)}^{\prime },g\right)$ and verifies whether $K_{1}^{*}=K_{0}^{*}\cdot K_{2}^{*}\cdot K_{3}^{*}\cdot K_{4}^{*}$ holds. If this equation holds, then **SA-Verify**(*pp*, *V String*, ID*, t*, M*, σ*) = 1.

In the following, we analyse the probability that the invalid message-signature pair $\left(M^{*},\sigma^{*}=\left(\sigma_{1}^{*},\sigma_{2}^{*},\sigma_{3}^{*},\sigma_{4}^{*}\right)\right)$ satisfies the condition $K_{1}^{*}=K_{0}^{*}\cdot K_{2}^{*}\cdot K_{3}^{*}\cdot K_{4}^{*}$. Since (σ*)′ = (σ*)^x*^ and x* is randomly chosen by C from $Z_{p}^{*}$, the probability that A can derive x* from (σ*)′ is $\frac{1}{p-1}$. As $x^{*}\in Z_{p}^{*}$, the probability of finding an integer x* that satisfies the condition $K_{1}^{*}=e{\left(g_{2},g_{1}\right)}^{x^{*}}\cdot K_{2}^{*}\cdot K_{3}^{*}\cdot K_{4}^{*}$ is also $\frac{1}{p-1}$. Moreover, because *p* is a large prime, A can convince C that σ* is a valid signature for message M* on (ID*, t*) with a negligible probability ($\frac{1}{p-1}$). Therefore, the algorithm **SA-Verify** in our SA-RIDPRS scheme satisfies the soundness requirement.

By combining Theorem 1, Lemma 3 and Definition 4, the following theorem can be deduced.

**Theorem 2.** In the standard model, our SA-RIDPRS scheme is secure against adaptive chosen message and collusion attacks.

### Performance analysis

In this section, we discuss the performance of our schemes and other IDPRS schemes in the standard model in terms of their computational cost and security properties. Because multiplication and hash function operations have relatively low computational costs, we consider only bilinear pairing and exponentiation operations, which have relatively high computational costs. In Table 1, “*Size*” denotes the size of a signature; “*Sign*”, “*Verify*” and “*ReSign*” denote the computational cost of the **Sign**, **Verify** and **ReSign** algorithms, respectively; and “*Revocation*” denotes whether the scheme includes a user revocation function. *E* represents the time to execute an exponentiation operation; *P* represents the time to execute a bilinear pairing operation; |*p*| represents the length of an element in *Z*_*p*_; and |*G*_1_| represents the length of an element in group *G*_1_.

**Table 1:** Performance comparisons with previous schemes.

Scheme	Size	Sign	Verify	ReSign	Revocation
Shao et al.’s scheme [20]	3|*G*_1_|	2*E*	3*P*	2*E* + 3*P*	No
Feng et al.’s scheme [21]	3|*G*_1_|	3*E*	3*E* + 5*P*	5*E* + 4*P*	No
Hu et al.’s scheme [22]	4|*G*_1_| + |*p*|	6*E*	4*E* + 3*P*	6*E* + 3*P*	No
Our RIDPRS scheme	4|*G*_1_|	2*E*	4*P*	2*E* + 4*P*	Yes
Our SA-RIDPRS scheme	4|*G*_1_|	2*E*	4*E* + *P*	6*E* + *P*	Yes

As shown in Table 1, in our SA-RIDPRS scheme, the verifier must perform one pairing operation and 4 exponentiations to verify the validity of a signature. In contrast, the verifier must compute 4 pairings in our RIDPRS scheme. Based on the results reported in [34], the computational overhead of a bilinear pair is, at minimum, more than five exponentiations in *G*_1_. Obviously, our SA-RIDPRS scheme is considerably less computationally intensive than is our RIDPRS scheme; therefore, it substantially reduces the verifier’s computational overhead, making it suitable for limited-resource devices.

In terms of signature size, the signature in our SA-RIDPRS scheme contains 4 elements in *G*_1_. The signatures in Shao et al.’s scheme [20] and Feng et al.’s scheme [21] contain 3 elements in *G*_1_, while the signature in Hu et al.’s scheme [22] contains 4 elements in *G*_1_ and one element in *Z*_*p*_. However, our two schemes are the only ones that provide user revocation functionality and can withstand signing key exposure attacks.

In the signing phase, our SA-RIDPRS scheme and Shao et al.’s scheme [20] require 2 exponentiations to generate a signature on a message, whereas the schemes of Feng et al. [21] and Hu et al. [22] require 3 and 6 exponentiations respectively.

In the re-signing phase, our SA-RIDPRS scheme performs one pairing operation and 6 exponentiations to create a valid re-signature, while the scheme of Shao et al. [20] requires 3 pairing operations and 2 exponentiations, and that of Feng et al. [21] must perform 4 pairing operations and 5 exponentiations. Hu et al.’s scheme [22] requires 3 pairing operations and 6 exponentiations.

During signature verification, the computational cost in our SA-RIDPRS scheme involves one pairing operation and 4 exponentiations. The schemes of Shao et al. [20] requires 3 pairings, and that of Feng et al. [21] requires 5 pairings and 3 exponentiations. Hu et al.’s scheme [22] requires 3 pairings and 4 exponentiations. In summary, our SA-RIDPRS scheme has considerably less computational overhead than do the other schemes.

**Fig 1:**
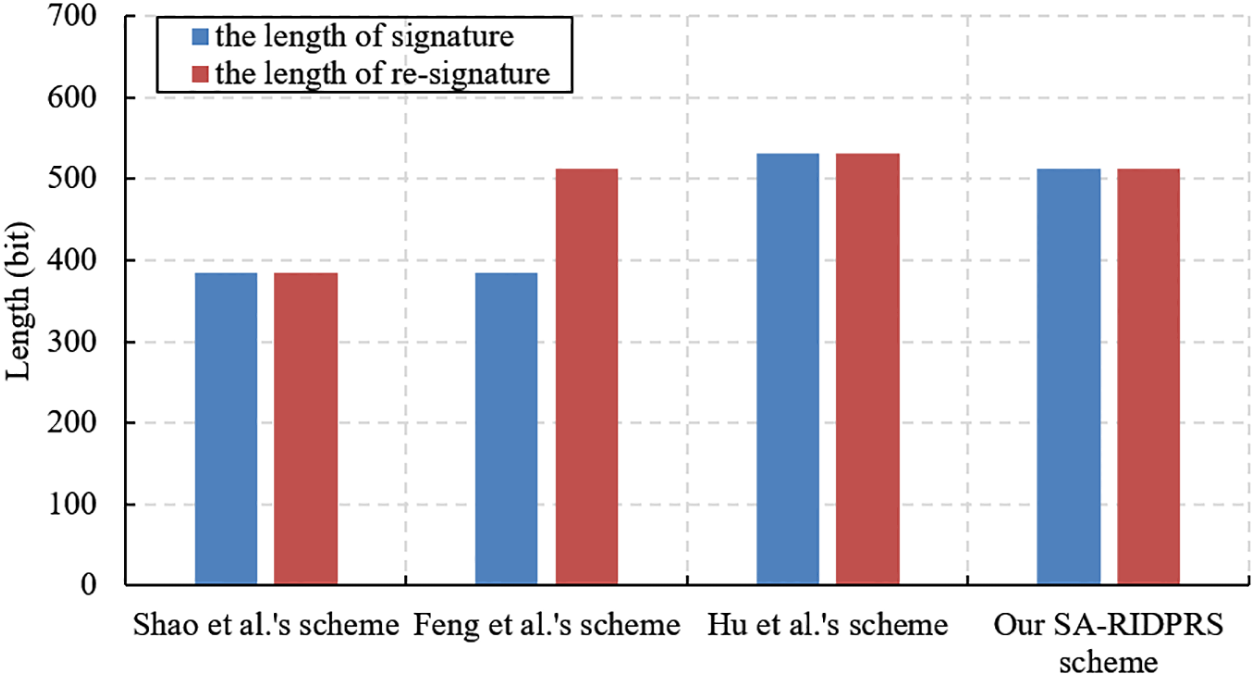
Comparison of signature/re-signature size.

**Fig 2:**
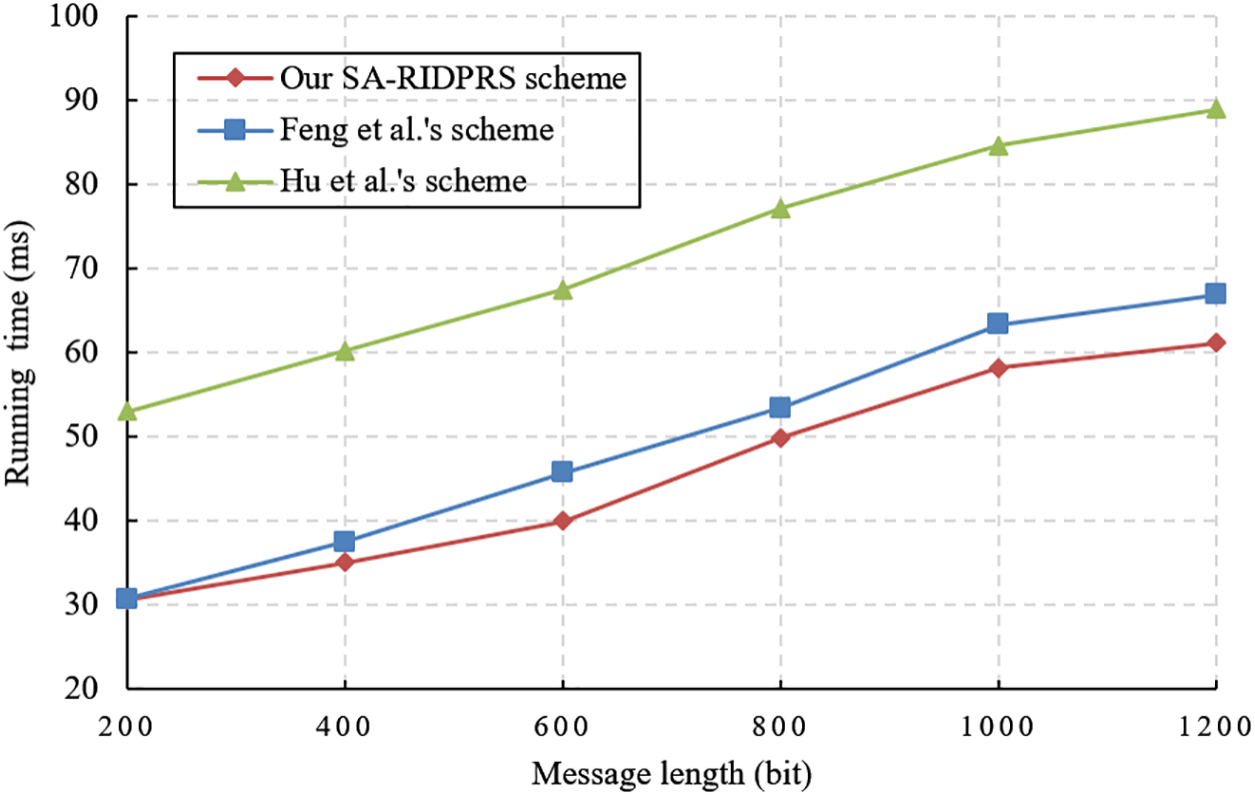
Signature generation cost comparison.

**Fig 3:**
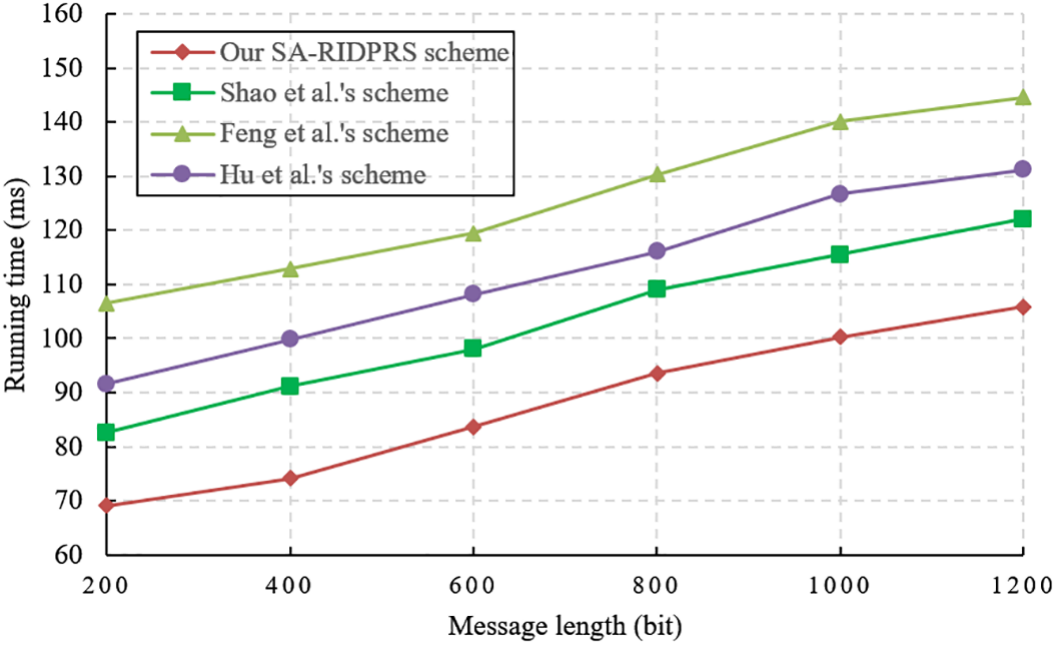
Re-signature generation cost comparison.

**Fig 4:**
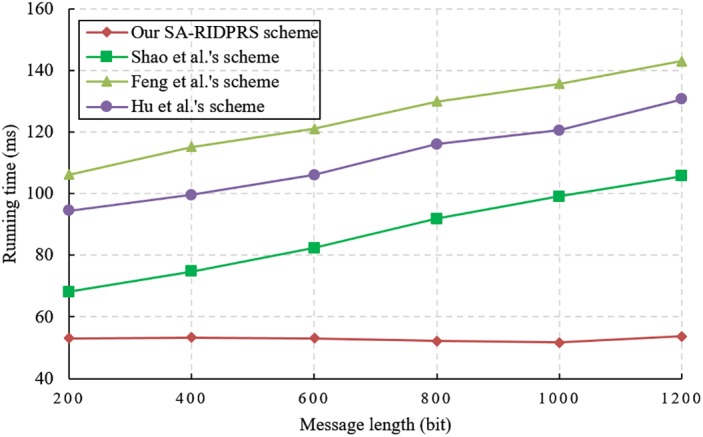
Verifier’s computational cost comparison.

We evaluate the performance of our SA-RIDPRS scheme and the three other IDPRS schemes [20–22]. The experiments are implemented in a hardware environment consisting of an Intel Core i7-6500 CPU 2.5 GHz with 8 GB of RAM. The corresponding simulation algorithms are implemented in the C language using the PBC-0.47-VC library and executed on a Windows 10 operating system. We chose the standard parameter *a*.*param* in the PBC library and set the group order *p* = 160 bits. The performance comparison results for all the schemes are shown in Figs 1, 2, 3 and 4.

In Fig 1, we can see that the length of the signature and re-signature in Shao et al.’s scheme [20] are both 532 bits. The length of the signature in Feng et al.’s scheme [21] is 384 bits, but the length of the re-signature is 512 bits. The length of the signature and re-signature in Hu et al.’s scheme [22] are both 532 bits. The length of the signature and re-signature in our SA-RIDPRS scheme are both 512 bits. However, Feng et al.’s scheme [21] is not multi-use because the lengths of signatures and re-signatures are different. While our SA-RIDPRS scheme includes one more element in *G*_1_ than the scheme of Shao et al. [20] does, it also effectively solves the user revocation problem. Therefore, compared with the other three schemes, our SA-RIDPRS scheme has comparable efficiency in terms of signature size. This is consistent with the above theoretical analysis.

According to Table 1, the cost of generating a signature in our SA-RIDPRS scheme is equivalent to that of Shao et al.’s scheme [20]. The experimental results shown in Fig 2 indicate that our SA-RIDPRS scheme has lower computational overhead for signature generation than do the other two schemes [21, 22]. Fig 3 demonstrates that our SA-RIDPRS scheme improves on the other three schemes [20–22] in terms of the computational overhead for re-signature generation. From Fig 4, we can observe that the computational cost of the verifier is not related to the size of the message in our SA-RIDPRS scheme. Because the verifier in our SA-RIDPRS scheme offloads most of the time-consuming bilinear pairing computations to the server via the server-aided verification algorithm, it can perform signature verification at a relatively low computational cost. Thus, our SA-RIDPRS scheme greatly reduces the cost of the verifier. Moreover, our SA-RIDPRS scheme is more efficient than are the other schemes [20–22] in terms of the verifier’s computational overhead. Clearly, the results of these experiments are consistent with the results of the theoretical analysis in Table 1.

## Conclusions

In this paper, we present two IDPRS schemes that include a user revocation mechanism. In our schemes, the PKG constructs an identity-dependent long-term key for all users and periodically generates update keys related to their identities and time periods for non-revoked users. Only non-revoked users can re-randomize the secret key and the update key to generate a corresponding signing key; therefore, our schemes are resistant to signing key exposure attacks. In the standard model, we prove that our schemes are secure under the CDH assumption. In particular, our SA-RIDPRS scheme substantially reduces the verifier’s computational burden. However, the security of our schemes relies on the hardness assumption of the CDH problem, which can be easily solved by quantum algorithms. In the future, we plan to construct a post-quantum secure RIDPRS scheme.
